# Unlocking the Therapeutic Potential of Ellagic Acid for Non-Alcoholic Fatty Liver Disease and Non-Alcoholic Steatohepatitis

**DOI:** 10.3390/antiox13040485

**Published:** 2024-04-18

**Authors:** Tharani Senavirathna, Armaghan Shafaei, Ricky Lareu, Lois Balmer

**Affiliations:** 1 Centre for Precision Health, School of Medical and Health Sciences, Edith Cowan University, Perth, WA 6027, Australia; tharanis@our.ecu.edu.au; 2 Centre for Integrative Metabolomics and Computational Biology, School of Science, Edith Cowan University, Perth, WA 6027, Australia; a.shafaeidarestani@ecu.edu.au; 3 Curtin Medical School and Curtin Health Innovation Research Institute, Faculty of Health Sciences, Curtin University, Perth, WA 6845, Australia

**Keywords:** ellagic acid, urolithins, non-alcoholic fatty liver disease, non-alcoholic steatohepatitis, dietarily interventions

## Abstract

Obesity is in epidemic proportions in many parts of the world, contributing to increasing rates of non-alcoholic fatty liver disease (NAFLD). NAFLD represents a range of conditions from the initial stage of fatty liver to non-alcoholic steatohepatitis (NASH), which can progress to severe fibrosis, through to hepatocellular carcinoma. There currently exists no treatment for the long-term management of NAFLD/NASH, however, dietary interventions have been investigated for the treatment of NASH, including several polyphenolic compounds. Ellagic acid is one such polyphenolic compound. Nutraceutical food abundant in ellagic acid undergoes initial hydrolysis to free ellagic acid within the stomach and small intestine. The proposed mechanism of action of ellagic acid extends beyond its initial therapeutic potential, as it is further broken down by the gut microbiome into urolithin. Both ellagic acid and urolithin have been found to alleviate oxidative stress, inflammation, and fibrosis, which are associated with NAFLD/NASH. While progress has been made in understanding the pharmacological and biological activity of ellagic acid and its involvement in NAFLD/NASH, it has yet to be fully elucidated. Thus, the aim of this review is to summarise the currently available literature elucidating the therapeutic potential of ellagic acid and its microbial-derived metabolite urolithin in NAFLD/NASH.

## 1. Introduction

Non-alcoholic fatty liver disease (NAFLD) is the physiological manifestation of obesity in the liver. This fast-growing epidemic is the most prevalent form of chronic liver disease globally. The prevalence of NAFLD has increased from 25.24% in 2015 to 29.38% in 2021 [1,2,3]. This condition now accounts for 45.8% of all cases of chronic-liver-disease-related deaths worldwide [1,3,4]. NAFLD can advance to non-alcoholic steatohepatitis (NASH), and approximately 20% of the affected population is likely to develop NASH [1,5]. Hepatic steatosis, alongside hepatocellular injury and inflammation, define NASH as these are the significant contributors to the onset of cirrhosis and hepatocellular carcinoma [1,6]. Although the specific pathogenesis of NASH remains uncertain, associated risk factors such as excess caloric intake, sedentary lifestyle, insulin resistance, liver lipogenesis, and gut microbiota dysbiosis are well established [7,8,9,10]. Despite years of extensive global research, there is currently no approved drug for the treatment of NASH [5,10,11,12]. Oxidative stress significantly contributes to the progression from NAFLD to NASH evidenced by an increase in oxidative stress and impaired antioxidant defence mechanisms throughout disease progression [7,13]. Several studies have been undertaken to investigate the efficacy of antioxidants in mitigating this phenomenon [10,14,15,16]. Ellagic acid is widely recognized for its antioxidant properties, but it also exhibits anti-inflammatory, antifibrotic, and anticancer properties [17,18,19,20]. In recent decades, this common non-flavonoid polyphenolic compound caught the attention for its hepatoprotective properties and as a therapeutic agent for treating NAFLD/NASH [14,21,22]. This review focuses on the effectiveness of ellagic acid in treating NAFLD/NASH by summarising relevant literature concerning its potential therapeutic mechanism on the liver.

## 2. Current Understanding of NAFLD/NASH

NAFLD represents a range of conditions from simple fatty liver (non-alcoholic fatty liver, NAFL) to NASH, which can progress to more severe fibrosis, cirrhosis, and potentially liver cancer [9,23]. NAFLD is characterised by over-accumulation of triglycerides in hepatocytes and is actively involved in all aspects of the Metabolic Syndrome, including obesity, type 2 diabetes mellitus, arterial hypertension, and hyperlipidaemia [24,25,26]. Progression to NASH is characterised by active liver tissue damage through increased inflammation and hepatocyte ballooning, as measured by the NAFLD Activity Score (NAS) [6].

Research advocates have campaigned for the renaming of NAFLD to metabolic dysfunction associated with fatty liver disease (MAFLD), arguing that NAFLD is viewed as an exclusionary term, defined only in the absence of conditions such as viral hepatitis B and C, autoimmune disorders, or excessive alcohol consumption [27,28]. In contrast to NAFLD, MAFLD is diagnosed on the onset of hepatic steatosis alongside the presence of one of the following: obesity/overweight, diabetes mellitus, or indicators of metabolic dysregulation [29,30]. Globally, MAFLD affects 38.8% of adults, carrying the potential to progress to cirrhosis and instigate significant extrahepatic conditions such as cardiovascular disease and chronic kidney disease [31,32]. However, the diagnostic criteria and the concept are novel and yet to be further tested and validated.

### Manifestation of NAFLD/NASH

The current understanding of NAFLD/NASH development and progression has shifted from the traditional two-hit hypothesis to a multiple, parallel-hit hypothesis, where pathogenetic influences act synergistically [8]. According to the multiple-hit hypothesis, hepatocellular damage not only originates from insulin resistance but also from dysbiosis of the gut microbiota, overnutrition, secretion of hormones from adipose tissue, and genetic and epigenetic factors [7,8]. Insulin resistance plays a pivotal role in altering lipid metabolism in the body, where it stimulates hepatic de novo lipogenesis and adipose tissue lipolysis, causing an escalated influx of fatty acids to the liver [33,34,35,36]. This results in elevated levels of inflammatory cytokine and adipokine secretion due to adipose tissue dysfunction [34,37]. Adipokines such as adiponectin and leptin induce insulin resistance, stimulate lipogenesis, and trigger inflammation, resulting in the accumulation of triglycerides within hepatocytes and their damage [38,39,40]. Cytokines play a crucial role in promoting inflammation in the liver. Pro-inflammatory cytokines such as tumour necrosis factor-alpha (TNF-α), interleukin-6 (IL-6), and interleukin-1 β (IL-1β) activate and enhance liver inflammation, leading to hepatic inflammation and hepatocyte damage [41,42,43]. Intrahepatic accumulation of fatty acids induces the production of lipotoxic lipids, which then increase endoplasmic reticulum stress and mitochondrial dysfunction. More importantly, in the liver, this results in an increase in oxidative stress and inflammation, causing the progression of NASH from steatosis [11,44,45]. Dysbiosis of the gut microbiome has also been found to activate the innate immune system, causing a cascade of inflammatory cytokines to be released from immune cells [46,47,48,49,50]. Subsequently, the gut microbiome has been identified as a key player in the progression of NAFLD to NASH; however, further research is needed to fully understand the role of the gut microbiome. One such mechanism of action is dysbiosis of the gut microbiome, which alters bile acid production and composition [51,52,53]. Alterations in bile acids can damage hepatocytes by promoting cellular injury, apoptosis, mitochondrial dysfunction, membrane disruption, and oxidative stress leading to liver damage [54,55,56]. These interactions further add to the hepatocellular damage, propelling the progression of NAFLD to NASH [7,8,9,57].

An increase in oxidative stress has been identified to be a major accelerant in the progression of NAFLD to NASH [58,59]. Malfunction of cellular organelles, such as the mitochondria and endoplasmic reticulum drives the increase in oxidative stress seen with liver damage [44,60]. During the progression of liver disease, uncontrolled accumulation of free fatty acids and lipotoxic lipids leads to an increase in reactive oxygen species and the exhaustion of the antioxidant defence system, which includes both enzymes and non-enzymatic antioxidants in the liver, which in turn may cause changes in hepatocyte structure and function [61,62]. Collectively, these processes result in chronic inflammation followed by hepatocyte cell death and hepatic stellate cell (HSC) activation, which then leads to fibrosis [45,59,60,63,64]. At the cellular level, the development of NASH is characterised by interactions between resident liver cells and immune-recruited cells, including liver progenitor cells (LPC), HSCs, and macrophages [26,65,66,67,68].

## 3. Current Treatments

There are no specific medications approved by the European Medicines Agency (EMA) or the Food and Drug Administration (FDA) for the treatment of NAFLD/NASH [10,12,69,70]. Lifestyle changes that include healthier eating and regular exercise are an effective treatment for NASH [71,72], along with weight loss and bariatric surgery [10,65,71,73,74,75]. Increased intake of carbohydrates, trans- and saturated fats, animal proteins (red meat), and processed food coupled with low intake of fibrous food are linked with NAFLD/NASH progression [76,77]. Portion control and calorie restriction are important, as excessive calorie consumption can lead to weight gain and exacerbate liver fat accumulation [78,79]. Recommendations suggest focusing on whole grains and low-glycemic index foods for carbohydrate intake and prioritising monounsaturated and polyunsaturated fats over other types for dietary fat intake [77,79,80,81,82]. Additionally, integrating vegetable protein sources, prebiotic fibers, and probiotic-enriched foods into the diet not only aids in reducing calorie intake but also fosters healthy gut microbiota [76,83,84]. Moreover, incorporating antioxidant-rich foods like berries, nuts, and leafy greens can mitigate oxidative stress and inflammation, crucial factors contributing to liver damage in NAFLD/NASH [78,84,85]. However, unlike communicable diseases, these treatments experience high dropout rates, a common phenomenon observed with other therapies that require individual behavioural changes. The dropout rate for lifestyle-based treatments, including diet change and greater physical activity has been reported to be between 10–80% [86]. These findings illustrate the necessity for effective treatments utilising pharmacological agents, both as a complement to lifestyle modifications and as a standalone approach for managing the significant proportion of patients that are unable to adopt “healthier” lifestyles due to mental and physical impairments, as well as busy lifestyles.

Several therapeutic approaches have been proposed for the treatment of NASH, however, none have yet been approved [10,12,87,88]. Recent studies indicate that antidiabetic drugs and glucagon-like peptide 1 (GLP-1) receptor agonists represent promising treatments for NASH [89,90]. Two types of agonists, Liraglutide and Semaglutide, have been shown to be effective in reducing insulin resistance, liver lipotoxicity, and hyperglycemia in patients with NASH [89,91,92]. However, a lack of oral administration and an increased risk of pancreatitis are two known side effects of these drugs [12].

Semaglutide, an FDA-approved treatment for type 2 diabetes, is recognised for its greater metabolic effects compared to Liraglutide. In a randomized, placebo-controlled, phase 2 clinical trial, researchers investigated Semaglutide impact on the histologic resolution of NASH in patients diagnosed with biopsy-confirmed NASH and fibrosis [93]. The findings revealed that a significantly higher percentage of patients experienced NASH resolution with Semaglutide compared to the placebo (59% with the 0.4-mg dose vs. 17% with the placebo), but there was no significant difference observed in improving fibrosis [93]. The study is currently progressing to the next phase, with an ongoing phase 3 trial (NCT04822181). Several clinical trials have shown that peroxisome proliferator-activated receptor (PPAR)-γ agonists, such as pioglitazone, significantly reduce steatosis, inflammation, and fibrosis, whilst improving levels of plasma alanine aminotransferase (ALT) and aspartate aminotransferase (AST) [94,95,96,97]. However, side effects such as weight gain, bone loss in women, and possible bladder cancer impede the use of these drugs in the treatment of NASH [95]. Lanifibranor acts as an oral pan-PPAR agonist. A successfully conducted 2b double-blind, randomised, placebo-controlled clinical trial on Lanifibranor [98] identified that patients administered a once-daily dose of 1200 mg demonstrated a significantly higher reduction percentage (55% vs. 33% for placebo) in the SAF-A score (representing the activity component of Steatosis, Activity, Fibrosis) with no exacerbation of fibrosis compared to the placebo group [98]. The study has progressed to the phase 3 trial (NCT03008070).

Recent findings suggest that vitamin E treatment may be more suitable for paediatric patients with NASH compared to adults [99], targeting oxidative stress and inflammation [95,100]. However, long-term use of vitamin E has been associated with increased risks of prostate cancer and haemorrhagic stroke [101,102,103]. In a randomised, controlled trial spanning two years, non-diabetic patients with NASH were administered either vitamin E (800 IU, natural form, once daily), pioglitazone (30 mg once daily), or a placebo, revealing that vitamin E improved NASH in 43% of patients, while pioglitazone showed no significant effect compared to placebo. Currently, both pioglitazone and vitamin E are used on a case-by-case basis, as a comprehensive understanding of the risk factors is yet to be thoroughly understood [2,95,104].

Resmetirom has recently emerged as a promising candidate for the treatment of NAFLD/NASH. As an agonist for the thyroid hormone receptor-β, Resmetirom aims to address key metabolic pathways compromised during the progression of NAFLD/NASH, including lipid metabolism, fibrosis, and inflammation [105]. Results from a randomised, double-blinded, placebo-controlled phase 2 clinical trial demonstrated significant efficacy of resmetirom compared to placebo over 12 and 36 weeks. Adult patients exhibited a significantly greater relative reduction in hepatic fat (32·9% with resmetirom vs 10·4% with placebo) as assessed by liver biopsy [106]. The most recent randomised, double-blinded, placebo-controlled phase 3 clinical study (NCT03900429) corroborates these findings, indicating that resmetirom is safe and well-tolerated in NASH patients over 52 weeks [107].

## 4. Ellagic Acid and Dietary Sources

Ellagic acid (EA) is a polyphenolic, non-flavonoid compound naturally found in a variety of fruits, such as pomegranates, raspberries, strawberries, and grapes, and nuts, such as pistachios, pecans, walnuts, and acorns [17,21,108,109]. Ellagic acid is a dilactone with the chemical name 2,3,7,8-Tetrahydroxy [1] benzopyrano [5,4,3-cde][1]benzopyran-5,10-dione (C_14_H_6_O_8_; MW: 302.194 g/mol; CAS number, 476-66-4) possessing both a hydrophilic moiety with four hydroxyl groups and two lactone groups together with a lipophilic moiety with two hydrocarbon rings [110]. This structure enables EA to accept electrons from several substrates, thereby participating in antioxidant redox reactions [110,111]. EA is commercially available as a nutraceutical product and known to attenuate chronic diseases such as metabolic syndrome, cardiovascular disease, hypertension, and neurodegenerative diseases [112,113,114,115].

### 4.1. Ellagic Acid Metabolites

Dietary EA is presented in free form or as a hydrolysable complex polymer called ellagitannins, which can be further metabolized to release free EA and gallic acid [18]. Free EA is primarily absorbed by the stomach and small intestine. The remainder is either absorbed by the large intestine [17,116,117] or metabolised by the resident gut microbiota to produce a microbial-derived metabolite known as urolithin [18,118,119]. In the liver, EA undergoes phase I metabolism, which involves oxidation and hydrolysis followed by phase II metabolism [18]. During phase II metabolism, ellagic acid undergoes a series of reactions, including glucuronidation, sulfation, and methylation. This results in the formation of ellagic acid glucuronides, methyl ellagic acid glucuronides, and dimethyl ellagic acid glucuronide, which are found in bile, confirming enterohepatic circulation [18,118,120,121].

**Figure 1:**
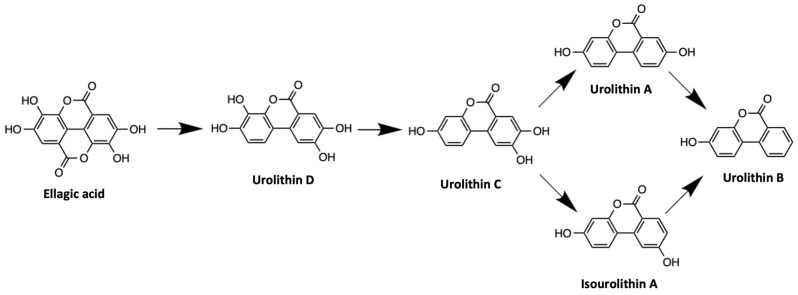
Chemical structures of ellagic acid and the formation of the gut microbial metabolite urolithins.

Ellagitannins are resistant to acid hydrolysis and, as such, are not directly absorbed in the stomach [122,123]. Hydrolysation and absorption of ellagitannins occurs in the small intestine, under a slightly basic or natural pH, which enables further absorption of free EA. EA is then absorbed across the gut epithelium through passive diffusion [17,124]. Unabsorbed free EA and ellagitannins are further metabolized and absorbed in the later part of the gastrointestinal tract. Urolithins are produced by gut microbiota during the metabolism of EA in humans [125,126] and several animals including rats [117] and mice [127,128]. Urolithin is a dibenzopyran-6-one derivative, which is also considered a benzo coumarin or dibenz-œ-pyrone [129]. During the microbial metabolism of EA, urolithin D is first produced via lactone ring cleavage and decarboxylation. Then, urolithin D is dehydroxylated from urolithin C, urolithin A, isourolithin A, and urolithin B, respectively (Figure 1) [17,20,129]. There are three main human metabotypes of urolithin, namely, metabotypes 0 (no urolithin production), metabotypes A (urolithin A), and metabotypes B (urolithin B, isourolithin A, and urolithin A) [119]. However, a study by Garcia-Villalba and colleagues revealed a new urolithin metabolic branch named R [130]. EA metabolism and urolithin production vary according to host health, age, gut microbial composition, environmental conditions, and metabotypes [14,119,131,132]. The presence of specific bacterial genera such as *Gordonibacter* and *Ellagibacter* has been shown to be necessary for the conversion of EA into urolithins [118,119,133,134]. Two gut microbial species, *Gordonibacte urolithinfaciens* and *Gordonibacte pamelaeae*, can produce urolithin C, whereas *Ellagibacter isourolithinfaciens* produces isourolithin A [132,135,136].

### 4.2. Bioavailability of Ellagic Acid, Ellagitannins, and Urolithins

The low bioavailability of ingested EA and its metabolites makes it difficult to detect and fully understand its metabolism. The level of EA in serum is very low (~200 ng mL^−1^) [137] but urolithins and their derivatives are present in micromolar levels, ranging from 0.024 to 35 μM in human plasma [118,120]. One glass of pomegranate juice (237 mL) can yield up to 300 mg of ellagitannins or about 120 mg of EA [138]. Around 100 g of raspberries produces 300 mg of ellagitannins and one strawberry yield up to 70 mg of ellagitannins [139]. However, urolithins have a greater absorption value than EA, possessing a higher lipo-solubility than free EA and, therefore, are more readily absorbed [17,140,141]. A study that investigated the metabolites present in defatted walnut powder administered to male Sprague Dawley rats revealed the presence of urolithin M5, urolithin C, and urolithin D [142]. Urolithin M5 is the first urolithin formed by the opening of one of the two lactone rings of ellagic acid, followed by decarboxylation [118,130]. These compounds were identified as hydrolyzation and dehydroxylation products of EA following the administration of defatted walnut power extract at a dosage of 10 g/kg for two days [142]. Interestingly, the study also detected both methylated and demethylated metabolites of EA in urine and faeces samples collected from the rats [142]. Analysis of blood and urine samples across several clinical studies indicated that the maximum concentration of EA occurred within 1–2 h and 0–4 h following gastrointestinal administrating [21,137,138,143]. This rapid elimination of EA presents a significant challenge in comprehending its biological functions, biotransformation, and bioavailability in animal and human systems. Consequently, measuring EA and its metabolites allows researchers to identify potential targeted biomarkers and gain a deeper understanding of EA biological activity.

## 5. Mechanisms of Action

**Figure 2:**
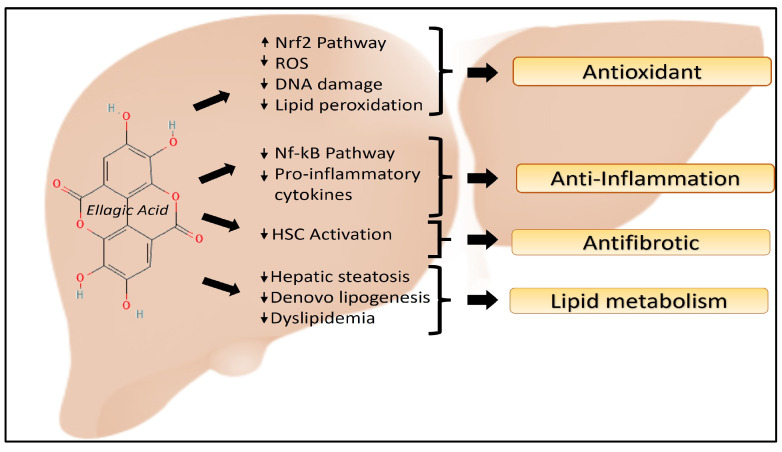
Positive impact of Ellagic Acid demonstrated through an increase of the Nrf2 pathway and decreasing all other pathways and mechanisms to alleviate NAFLD/NASH.

Ellagic acid and its microbial metabolite, the urolithins, have been reported to possess many beneficial pharmacological properties such as antioxidant, anti-inflammatory, antimutagenic, antidepressant, cardio-protectant, anticarcinogenic, and most of most relevance to this review, hepatoprotective activity (Figure 2) [18,22,109,144].

### 5.1. Oxidative Stress

EA is a recognised antioxidant with implications in modulating various molecular targets and pathways involved in numerous chronic diseases, including liver diseases [17,109,145]. With its four hydroxyl and two lactone functional groups, EA exhibits the capacity to scavenge a wide variety of free radical species, including reactive oxygen species (ROS) and reactive nitrogen species (NOS); thus, protecting against free radical-induced damage [145,146,147,148].

The presence of hydroxyl and peroxyl radicals enables the initiation and multiplication of lipid peroxidation, respectively. Studies have demonstrated the efficacy of EA in mitigating lipid peroxidation even at minimal concentrations, such as µM levels, which is primarily attributed to its potent free radical scavenging properties [149]. Interestingly, an earlier investigation proposed that EA was a superior free radical scavenger to vitamin E [150]. Recently, it has been reported that EA inhibited 71.2% of lipid peroxidation, surpassing the inhibition levels observed with well-established antioxidants, ascorbic acid and α-tocopherol (i.e., vitamin E), resulting in 64.5% and 59.7% inhibition, respectively [151]. According to Yu and colleagues [152], EA supplementation in rabbits fed an atherogenic diet led to significant improvements in their lipid profiles and a reduction in lipid peroxidation. This intervention also resulted in the suppression of 8-oxo-2′-deoxyguanosin (8-(OH)dG) levels, in addition to the expression of caspase-8, caspase-9, and Fas ligand in the aortic arch. These findings suggest that the reduction in lipid peroxidation contributes to the antioxidant effects of EA.

The presence of Ionic cations such as Zn^2+^, Ca^2+^, Fe^2+^, Cd^2+^, and Cu^2+^ can promote oxidation rates in a biological system. However, phenolic compounds such as EA can inhibit this process due to its chelating property, where, it binds to the cation and forms a complex that prevents oxidation [153,154]. Ahmed and colleagues [155] have previously described how EA (500 µmol/Kg BW(body weight)) has a chelating effect that suppresses nickel-induced oxidative stress in female Wistar rats. Furthermore, Kilic and co-authors [151] have reported that EA has a similar chelating effect as caffeic acid on ferrous ions. Moreover, EA and epigallocatechin gallate have a similar binding capability to iron, where this binding is enabled due to the presence of catechol groups in the former [156]. EA also can chelate copper and form stable complexes after deprotonation [157]. Collectively, these data demonstrate that EA is protected from oxidative stress by chelation.

The formation of 8-(OH)dG is a critical step in oxidative damage on DNA, and several studies have reported that EA drastically reduces 8-(OH)dG formed by oxidative DNA damage [158,159]. Previous in vitro studies have described this protective effect of EA via its ability to regulate intracellular mechanisms through direct interaction between double-strand DNA and EA [160,161]. An in vitro study by Spencer and colleagues [162] has revealed that EA has the ability to inhibit dopamine/Cu^2+^-induced oxidative DNA damage even at very low doses, as low as 1 µM. This suggests EA’s potential protective mechanism against oxidative stress through shielding DNA damage.

Nuclear factor erythroid 2–related factor (Nrf2) is a cellular antioxidant regulator that activates during cellular stress to induce genes related to the antioxidant defence system. Activation of these types of responses or signalling pathways plays a major role in protective mechanisms against oxidative stress [163,164]. In this regard, EA administration to high-fat diet (HFD)-fed apolipoprotein E-knockout mice (ApoE−/−), resulted in a reduction in oxidative stress and atherosclerosis via induction of the Nrf2 signalling pathway [165]. In vitro research conducted by Baek and colleagues [166] reported that EA upregulates the Nrf2 pathway in human dermal fibroblasts, this then plays a protective role against induced oxidative stress. Furthermore, another study by Gu and co-authors [167] has described the protective effect of EA in acute hepatic injury in mice, by inducing Nrf2 expression and heme oxygenase-1. These studies conclude that EA has protective properties against oxidative stress through upregulation of the Nrf2 pathway.

### 5.2. Inflammation

In addition to its well-known antioxidant properties, EA has been reported to possess anti-inflammatory properties [168]. As mentioned above, both oxidative stress and inflammation are closely associated [169,170]. Nuclear factor kappa B (Nf-kB) is a key transcription factor for proinflammatory responses, produced in all cell types and activated under several types of cell stress induced by obesity, oxidative stress, hyperglycaemia, hypertension, and bacterial infections [171,172,173]. A study by Ahad and colleagues [174] has reported EA supplementation attenuated dyslipidaemia and nephropathy in type 2 diabetic male Wistar rats. The authors described how EA ameliorated diabetic nephropathy by inhibiting the expression of the Nf-kB pathway. Nf-kB has also been reported to play a key role in the regulation of cyclooxygenase-2 (COX-2) expression, which is involved in the inflammatory process during tumour growth [175]. Administration of EA (100 mg/Kg BW) can modulate COX-2 mRNA, mainly through downregulation of ROS production, which in turn inhibits Nf-kB activation [176].

Different kinds of proinflammatory cytokines, such as macrophages and migration inhibitory factor (MIF), play a crucial role in facilitating an immune response [109]. It has been shown that MIF induces Nf-kB and chemotaxis during an inflammatory response [109,177,178,179]. EA (50 µM) downregulated the tautomerase activity of MIF and MIF-mediated proinflammatory responses in peripheral blood mononuclear cells [179]. EA also suppresses the expression of pro-inflammatory cytokines TNFα, IL-6, and chemokine C-C, in lipopolysaccharide (LPS)-stimulated macrophages and adipocytes, suggesting that EA may attenuate inflammation in adipose tissue [180]. Moreover, EA can significantly inhibit TNFα and IL-6 in LPS-stimulated RAW 264.7 cells, even with minimal µM concentrations (6.25 µM and 12.5 µM) [181]. Even though the exact mechanism of EA involvement in proinflammatory cytokine modulation is not clear, it has been proposed that this polyphenol acts through direct inhibition of the Nf-kB pathway.

Another mechanism of interest is the effect of EA on resistin, an adipocytokine which may be the missing link between obesity and type 2 diabetes [182,183]. Pomegranate fruit extract suppressed enhanced levels of serum resistin in mice [184]. The authors also showed that EA reduced resistin levels in 3T3-L1 cells in vitro. Another study by the same authors demonstrated that EA reduced serum resistin levels without changing mRNA expression in adipose tissue. Further to this, they demonstrated that EA significantly improved hepatic steatosis and serum lipid composition in KK-A^y^ mice, thus further contributing to EA in the suppression of resistin secretion in vivo [185].

## 6. Therapeutic Efficacy

Recent studies, both in vivo and in vitro, have shown that EA forms several natural metabolites that have potential therapeutic properties for chronic diseases such as liver disease [18,21,109]. Research has focused on this nutraceutical due to its pharmacokinetic properties, safety, and efficiency [109,186,187]. A study conducted to determine the role of muscadine grape extract vs muscadine grape wine in obesity has demonstrated that both supplementations reduced plasma triglycerides, free fatty acids, and cholesterol levels [188]. However, muscadine grape extract also demonstrated a higher lipid-lowering effect on both triglycerides content and adipose tissue mass and possesses a higher content of EA (18 mg EA/Kg BW) than the wine. The wine, which has similar polyphenolic content but lower EA content (1.1 mg EA/Kg BW), demonstrated lower metabolic improvements, suggesting EA might play a crucial role in improving the Metabolic Syndrome.

The involvement of gene regulation in fatty acid oxidation has been studied by Cao and colleagues [189], demonstrating that supplementation of punicalagin (150 mg EA/Kg BW), one of the main ellagitannins in pomegranate, attenuated HFD-induced obesity in rats through mechanisms involving AMP-activated protein kinase (AMPK) activation [189]. The AMPK pathway is known to be involved in the regulation of energy homeostasis via inhibiting de novo lipogenesis and adipogenesis [190]. Raspberry seed flour supplementation (100 mg EA/Kg BW) in a high sucrose diet mouse model has been used to demonstrate a reduction in hepatic endoplasmic reticulum stress, dyslipidemia, and adipose tissue inflammation, supporting the role of EA in preventing sugar toxicity [191]. Panchal and colleagues suggested EA supplementation (80 mg EA/kg BW) to be beneficial to improving the liver structure and function of HFD-induced male Wistar rats via blunting oxidative stress and inflammation. Administration of EA supplementation in the above study reduced the Metabolic Syndrome by regulating the protein levels of Nrf2, Nf-kB, and carnitine palmitoyl transferase-1 to their basal levels [108]. Further to this, EA has been proposed as a therapeutic nutrient for NAFLD. This was demonstrated when large lipid accumulation in HFD-induced liver disease was eliminated by punicalagin-enriched pomegranate extract (150 EA mg/kg/day) treatment in a rat model, where a marked reduction in liver triglyceride and cholesterol levels was observed [61]. Defatted walnut power extract contains many active polar substances including EA [192,193,194]. Ren and colleagues [195] have conducted a study investigating the anti-NAFLD effects of this walnut extract on an HFD-induced mouse model. The results showed that defatted walnut power extract reduced the expression of Nf-kB and the mitogen-activated protein kinases (MAPK) family, thus inhibiting inflammation in the liver during disease progression. This study also demonstrated that the administration of defatted walnut power extract improved the gut microbiota diversity disrupted by NAFLD [195]. A more recent study demonstrated that EA ameliorates high fructose-induced hyperuricemia and NAFLD through activation of C1q/tumour necrosis factor-related protein (CTRP3) and inhibition of ATP citrate lyase (ACL) in male albino rats [196]. Hyperuricemia occurs due to the damaged metabolism of uric acid [197] and is attributed as a risk factor for NAFLD [198,199,200].

New insights into research on EAs have focused on its microbial metabolites, the urolithins. According to Abdulrahman and colleagues [133], supplementation of urolithin A to HFD-fed Wistar rats, reduced bodyweight, serum levels of cholesterol and low-density lipoprotein (LDL), and increased levels of high-density lipoprotein (HDL). Moreover, this study demonstrated that urolithin A administration reduced the abundance of the specific microbiome related to weight gain, dysfunction of lipid metabolism, and impaired glucose metabolism, suggesting urolithin A has the potential to act as a therapeutic agent in obesity [133]. A study on both EA and urolithin A has revealed that in a high-fat/high-sucrose-fed mouse model, only EA demonstrated the capability to reduce proton leakage in primary hepatocytes, as opposed to urolithin A; thus, reporting its involvement in mitochondrial respiratory capacity during insulin resistance [201]. Mitochondrial proton leak can lead to impaired insulin signalling pathways, contributing to the development of insulin resistance, a key factor in NAFLD progression [44,202,203]. Intragastrical administration of urolithin A (50 or 100 mg/kg per day) improved hepatic steatosis induced by fructose consumption in a high-fructose-fed mouse model [204]. The results also stated that urolithin A inhibited lipogenesis while enhancing an increase in β-oxidation in the liver. Additionally, it promoted hepatic lipophagy through the AMPK/ULK1(Unc-51-like kinase 1) pathway. The AMPK/ULK1 pathway serves as a key regulator of lipid metabolism and autophagy within liver cells [205]. This suggests that AMPK/ULK1 regulates hepatic lipophagy when stimulated by urolithin A, thereby being a potential therapeutic for the treatment of NAFLD.

Xu and colleagues [206] in a recent study, used oral administration of urolithin C on a choline-deficient amino acid-defined high-fat-diet (CDAHFD) mouse model and demonstrated significantly improved liver index (weight of liver/body) and NAS score compared to the control and disease groups [206]. This study also revealed that the administration of urolithin C inhibited ferroptosis through activation of the hepatic AMP-activated protein kinase (AMPK) pathway [206]. Ferroptosis is a form of ion-dependent cell death and is recognised for its involvement in the pathogenesis of NAFLD. It is characterised by elevated oxidative stress and dysfunctional lipid metabolism [207,208]. Xu and colleagues also demonstrated that urolithin C ameliorated the permeability of the intestinal epithelial and increased the proportions of certain beneficial bacteria including *Parabacteroides goldsteinii* and *Lactobacillus vaginalis*, improving the dysbiosis caused by CDAHFD [206]. Another study conducted on Wistar rats on an HFD showed that both urolithins A and B improved characteristics associated with obesity, including weight gain, lipid accumulation, and oxidative stress [209]. The results of this study also revealed that both urolithins significantly downregulated the expression of liver X receptor (*LXRα*) and sterol regulatory element-binding protein-1c (*SREBP1c*) genes, which are involved in de novo lipogenesis. The authors showed that both urolithins A and B attenuate hepatic endoplasmic reticulum stress through the downregulation of unfolded protein responses [209].

The biological properties of EA have been investigated in several in vitro studies. One of the studies revealed that inhibiting HSC activation is highly sensitive to EA [210], suggesting its potential involvement in antifibrotic mechanisms. Furthermore, an in vitro study investigating the inhibitory effects of *Phyllanthus emblica* L. (Indian gooseberry) on hepatic steatosis and liver fibrosis identified EA as the main compound present in the water extract of *P. emblica* fruits [211]. In the study, a two-cell in vitro system to simulate NASH and hepatic fibrosis features, the authors used human hepatoblastoma HepG2 cells treated with a mixture of free fatty acids and the rat hepatic stellate HSC-T6 cells induced by leptin, respectively. The results showed the water extract reduced fat accumulation and ROS production through the modulation of the AMPK signalling pathway in HepG2 cells. They also demonstrated the potential for attenuating hepatic fibrosis in HSC-T6 cells and triggering mitochondrial apoptosis. Thus, indicating that EA has the potential to mitigate the progression of NAFLD [211].

Ellagic acid suppressed de novo lipogenesis by inhibiting the expression of sterol regulatory element-binding protein-1(SREBP-1)/fatty acid synthase (FASN) cascade and reducing steatosis in human hepatoma cell line through activation of AKT/mTORC1 (protein kinase B/mammalian target of rapamycin) in the liver [212]. Both SREBP-1 and FASN play a critical role in the increased production of fatty acids and exacerbating liver fat accumulation during NAFLD progression [213,214]. Dysregulation of the AKT/mTORC1 pathway can contribute to abnormal lipid metabolism, hepatocyte proliferation, and inflammation, ultimately promoting the development and progression [215,216]. Therefore, EA may hold therapeutic potential for managing NAFLD by targeting this pathway.

An in vitro study revealed that urolithin A promoted fatty acid breakdown, including both lipophagy and β-oxidation, via an AMPK-dependent pathway on fructose-treated HepG2 cells and primary hepatocytes. These in vitro experiments extended the findings from the high fructose-fed mouse model discussed above [204]. Additionally, a study conducted on oleic-acid-stimulated Alpha mouse liver 12 (AML12) cells revealed that urolithin C can activate the hepatic AMPK pathway to alleviate ferroptosis response in vivo but not in vitro [206]; thus, suggesting a crucial link between urolithin C and hepatic AMPK, likely through the gut-liver axis discussed earlier. Additionally, this study concluded that urolithin C was unable to reduce lipid accumulation or inhibit ferroptosis in vitro [206].

Consequently, in vitro findings suggest that both EA and urolithin possess the ability to regulate lipid metabolism during the progression of NAFLD. However, current data suggest that EA offers greater advantages compared to urolithin as it can inhibit both hepatic steatosis and hepatic fibrosis in vitro. Therefore, EA holds promise as a therapeutic agent for blunting the progression of the disease.

Despite a large amount of evidence being available in the form of pre-clinical studies, there are no clinical trials to date that have tested EA and its pharmacological properties on liver disease. A systematic review conducted by Gheflati and colleagues revealed that, even though many studies consider pomegranate as a tool to manage weight loss, there was no significant effect of pomegranate on body weight, BMI, and body fat percentage [217]. However, EA and pomegranate juice (naturally high in EA) have been evaluated in Phase I, II, and III clinical trials, primarily focusing on anticancer properties [218,219,220] and skin hyperpigmentation [221,222]. Even though there is a plethora of research on the potential hepatoprotective properties of EA, such as antioxidant and anti-inflammatory effects, most of these have not focused on Metabolic Syndrome, NAFLD, or diabetes.

## 7. Future Perspective of EA as a Pharmacological Therapy

EA stands out as a remarkable polyphenolic compound, possessing a wide range of pharmacological properties that hold promise in treating various chronic diseases, including NAFLD/NASH. Due to its multifaceted biological effects, edible plants containing EA and their derivatives are recognised as valuable functional foods for enhancing human health. Moreover, there is evidence suggesting that EA, when combined with other antioxidant nutraceuticals, exhibits a synergistic therapeutic effect, making it a potential candidate for combination therapy [113]. Although clinical trials investigating EA’s effects on NAFLD/NASH are pending, the pharmaceutical and cosmetic industries are already incorporating this polyphenolic compound into novel supplement preparations. Consequently, given the current widespread popularity of supplementation, it is important to consider incorporating EA as a dietary intervention for NAFLD/NASH. However, like many other polyphenols, the lack of comprehensive understanding regarding the underlying mechanisms governing its biological properties limits its capacity as a pharmacological agent in the market.

## 8. Conclusions

There is a great need for effective pharmacological treatments for NASH due to the severity, growing impact on the global health system, and more importantly, the absence of approved pharmacological treatments. EA exerts its hepatoprotective properties primarily through scavenging free radicals, modulating cytokine production, and regulating lipid metabolism. As an excellent antioxidant, EA acts against ROS and activates the NrF2 pathway to reduce oxidative stress to protect the liver. Remarkably, EA also suppresses Nf-kB and MAPK pathways, mitigating inflammation during NAFLD/NASH. The evidence also shows that EA can reduce both triglyceride and cholesterol levels, thus combating de novo lipogenesis, which is a significant risk factor in NASH progression. In vitro, findings suggest that EA has the capability to alleviate fibrosis. The primary microbial metabolite for EA, urolithin, has been shown to improve the gut microbiome in several mouse models of obesity. Specifically, urolithin A has been shown to lower LDL and increase HDL levels and is also involved in improving lipid metabolism through gene regulation, while urolithin C activates the hepatic AMPK pathway, thus counteracting the pathophysiology of NAFLD. There is an ongoing debate regarding the health benefits of EA and urolithins for NAFLD/NASH, but there is still a lack of understanding regarding their biological effect on the liver. Given the involvement of lipid metabolism, oxidative stress, inflammation, and insulin resistance in the pathogenesis of NASH, findings from this review suggest that EA may represent a potential food intervention for NASH, not only to limit but potentially reverse the pathological manifestations of NAFLD/NASH.
